# Beyond Oral Health: Personalized Strategies for Managing Oral Infections in Neutropenic Patients

**DOI:** 10.3390/jpm16010053

**Published:** 2026-01-16

**Authors:** Anca Elena Duduveche, Luminita Ocroteala, Adina Andreea Mirea

**Affiliations:** 1 Department of Infectious Diseases, University of Medicine and Pharmacy of Craiova, 200349 Craiova, Romania; 2 Department of Hematology, Municipal Clinical Hospital Filantropia Craiova, 200143 Craiova, Romania; diaconu_luminita@yahoo.com; 3 Department of Oro-Dental Prevention, University of Medicine and Pharmacy of Craiova, 200349 Craiova, Romania; adina.mirea@umfcv.ro

**Keywords:** neutropenic, oral infections, procedures, antibiotic stewardship, personalized medicine, risk stratification

## Abstract

Oral infections in neutropenic patients are an underestimated but likely fatal cause of infectious complications, with clinical manifestations often diminished or absent due to immune deficiency. The evaluation and management of these infections requires a personalized multidisciplinary strategy, including prevention through pre-therapy dental assessment, individualized oral hygiene protocols, and rapid treatment of dental lesions. Antimicrobial strategies should be adapted not only to the local resistance profile and individual risk, with a priority on antibiotic stewardship and rapid de-escalation when possible, but also to individual patterns of colonization and comorbidities. Dental procedures can be performed without risk in neutropenic patients with a low complication rate, but further studies are key to stratifying risk. Future research directions include the application of artificial intelligence for infectious risk stratification, the use of salivary or microbiome biomarkers for early detection, and the development of innovative technologies for targeted antimicrobial delivery. This narrative review aims to provide an overview of the common clinical manifestations in neutropenic patients and also the potential progression of dental infections into sepsis in this category of patients.

## 1. Introduction

Neutropenia is identified as a reduction in absolute neutrophil count (ANC), mostly resulting from chemotherapy, hematopoietic stem cell transplantation (HSCT) conditioning, aplastic anemia, chimeric antigen receptor (CAR) T-cell therapy, and immunosuppressant administration. Chemotherapy and HSCT are the most frequent causes, with CAR T-cell therapy and aplastic anemia also contributing to risk, particularly in hematologic malignancies and post-lymphodepletion settings [1,2,3,4].

Dental and oral infections are clinically significant in neutropenic patients because neutrophils are essential for mucosal defense. Neutropenia, especially when profound and prolonged, increases susceptibility to bacterial and fungal infections, and breaches in oral mucosal integrity (e.g., mucositis, periodontal disease) serve as portals for systemic infection. The American Society of Clinical Oncology and the Infectious Diseases Society of America underline that oral sources, among other things, mucositis and periodontal disease, can be overlooked but are significant contributors to febrile neutropenia and sepsis [1,4]. The diminished inflammatory feedback in neutropenia could possibly cover typical indicators of oral infection, augmenting the risk of undetected systemic dissemination [5].

Data on the prevalence of oral foci as sources of systemic infection indicate that oral lesions (ulceration, fungal, and viral infections) are standard issue in neutropenic patients, with oral ulceration spotted in up to 48% of chemotherapy-induced neutropenia cases [6]. Periodontal inflammation, quantified by the periodontal inflamed surface area (PISA), is per se associated with febrile neutropenia beginning in patients undergoing chemotherapy for hematologic malignancies [7]. Untreated odontogenic foci are a significant risk factor for odontogenic infection during neutropenia, regardless of neutropenia severity [8]. The infected periodontium can act as a focus for systemic infection, though the true incidence is likely underestimated due to subtle clinical presentation. Oral foci represent a significant and often underestimated source of systemic infection in neutropenic patients, and evaluation and treatment of oral lesions before initiation of chemotherapy or transplantation is recommended as part of supportive care.

Given the evolving landscape of supportive care in hematology and oncology, there is a need to integrate data from dentistry, infectious diseases, and hematology to better define how oral infections should be prevented, recognized, and managed in neutropenic patients. Therefore, the purpose of this narrative review is to provide a comprehensive overview of the epidemiology and clinical spectrum of oral infections in neutropenic patients (ulcerations, necrotizing gingivitis, periapical abscesses, mucositis-associated infections, and fungal superinfections), to discuss current diagnostic and therapeutic strategies through a personalized-medicine lens, and to highlight emerging approaches and knowledge gaps that may guide future research and clinical practice. Our objective was also to highlight the potential progression of dental infections to sepsis in this group of patients. This review employed a rigorous literature search and critical appraisal of studies focusing on oral infections in neutropenic patients, including observational studies, case reports, and reviews. We based on the search of publications that included the keywords “oral infection”, “dental”, “neutropenia”, “hematological malignancies”, “chemotherapy”, “oral infections in hematological patients”, “oral infections in hematological malignancies”, in PubMed, Scopus, Google Scholar and Web of Science databases. We focused on articles published between 2010–2025, but also included older articles when they were relevant.

Integrating dentistry, infectious diseases, and hematology allows clinicians to move beyond generalized protocols toward individualized risk stratification, targeted diagnostics, and personalized preventive or therapeutic interventions.

## 2. Pathophysiology

### 2.1. Impact of Neutrophil Depletion on Host Defense

Neutrophil depletion intensely compromises host defense mechanisms by disrupting the innate immune response, significantly against bacterial and fungal pathogens. Neutrophils are essential for phagocytosis, microbial eradication, and the regulation of inflammatory responses. In patients with neutropenia due to chemotherapy, hematopoietic stem cell transplantation conditioning, aplastic anemia, CAR-T therapy, or immunosuppressant use, the risk of infection multiplies with both the duration and severity of neutropenia [1,4].

This immunodeficiency is characterized by recurrent disruptions in mucosal barriers, such as oral mucositis and ulceration, which are generally met in these populations. The oral cavity, with its high microbial amount and susceptibility to mucosal injury, is a serious site for infection. Neutropenic patients often develop oral lesions—ulcers, fungal, and viral infections—at significantly higher rates, with oral ulceration described in almost fifty percent of patients with chemotherapy-induced neutropenia [6]. The eroded inflammatory response means that representative signs of infection (erythema, swelling, pus) may be absent, allowing local oral infections to progress undetected, thus advancing to systemic dissemination [5,6]. Periodontal inflammation, quantified by the periodontal inflamed surface area, is independently related to febrile neutropenia onset in patients going through chemotherapy for hematologic malignancy, highlighting the oral cavity as a frequent and under-recognized determinant of systemic infection [7]. Thus, neutrophil depletion not only increases susceptibility to oral infections but also facilitates their progression to life-threatening systemic infections due to impaired local and systemic immune surveillance.

### 2.2. Mechanisms of Progression (Figure 1)

Oral infections in neutropenic patients progress from gingivitis to periodontitis, subsequently to periapical infection, and ultimately result in bacteremia or sepsis due to loss of neutrophil-mediated guidance of oral biofilm pathogens and defective mucosal barrier integrity. In the absence of adequate neutrophil monitoring, gingivitis, which is initiated by biofilm accumulation at the gingival margin, quickly advances to periodontitis, characterized by deeper tissue invasion, ulceration of the pocket epithelium, and increased periodontal inflamed surface area. This breakdown permits oral pathogens and their products (e.g., lipopolysaccharide) to pass through the bloodstream, especially when the epithelial barrier is further compromised by chemotherapy-induced mucositis or ulceration [5,7,9]. As periodontitis evolves, infection can amplify to the periapical tissues, forming abscesses and further increasing the risk of systemic spread. In neutropenic states, the classic signs of inflammation are moderated, so these processes may be clinically silent until bacteremia or sepsis develops [10,11]. The risk of bacteremia is directly associated with the grade of periodontal inflammation and bleeding on probing [10].

**Figure 1:**
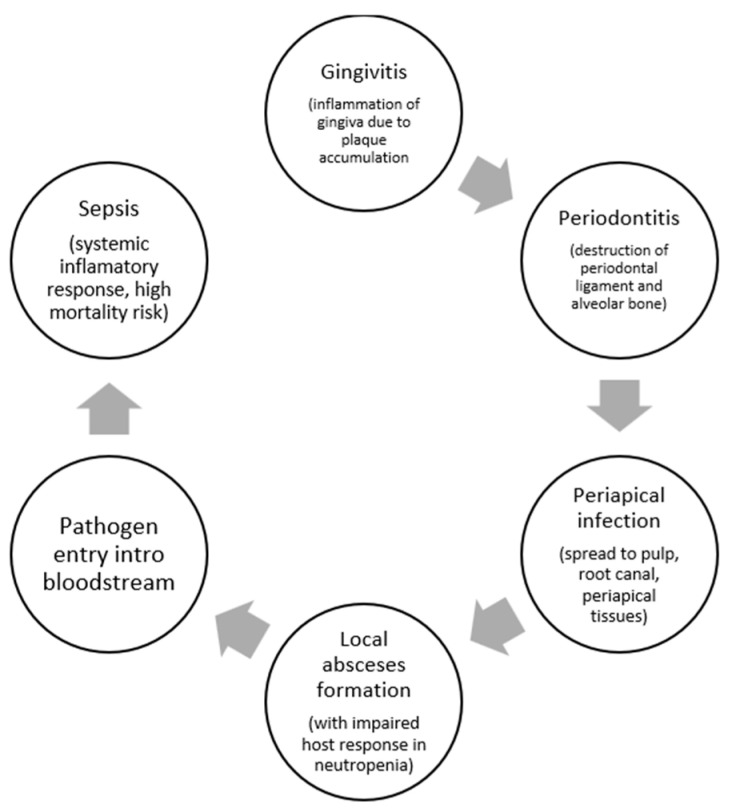
Progression from a minor dental lesion (caries, gingivitis, or early periodontitis) to rapid local infection, bloodstream invasion, and life-threatening systemic sepsis in the context of severe neutropenia.

### 2.3. High-Risk Oral Pathogens

Viridans group streptococci (VGS), especially *Streptococcus mitis* and *Streptococcus oralis*, are dominant oral commensals that become high-risk pathogens in neutropenic patients. They colonize dental plaque and gingival crevices, and in the setting of neutropenia, mucosal barrier injury (e.g., mucositis, periodontitis) facilitates their translocation into the bloodstream. VGS are the most frequent source of bacteremia in neutropenic patients, particularly after chemotherapy or hematopoietic stem cell transplantation, and are linked with severe complications, in particular septic shock and acute respiratory distress syndrome. The risk is increased by poor dental health, severe intraoral pathology, and antimicrobial prophylaxis with fluoroquinolones or trimethoprim-sulfamethoxazole, which can select for resistant strains. VGS bacteremia is typically endogenous, arising from the patient’s own oral flora, and is strongly associated with the presence of gingivitis or periodontitis and bleeding on probing, reflecting the role of periodontal inflammation in systemic spread [10,12,13,14,15].

*Enterococcus* species are not classic oral commensals, but can colonize the oral cavity, particularly in immunocompromised hosts. In neutropenic patients, *Enterococcus* can be isolated from oral lesions and is associated with mucosal inflammation and ulceration. These organisms may contribute to the progression from gingivitis to periodontitis and periapical infection, and can serve as a source of bacteremia, principally when the mucosal barrier is compromised [16,17].

*Pseudomonas* species, notably *Pseudomonas aeruginosa*, are environmental organisms that can colonize the oral cavity in neutropenic patients, especially those with severe periodontal disease or mucositis. *Pseudomonas* is detected at higher prevalence and abundance in the subgingival biofilm of patients with periodontitis and is associated with tissue destruction and increased risk of systemic infection. Oral colonization with *Pseudomonas* is a recognized risk factor for bacteremia in leukemia and transplant patients [17,18,19].

*Candida* species (especially *Candida albicans*) are frequent colonizers of the oral mucosa in neutropenic and immunosuppressed patients. *Candida* can proliferate in the context of mucosal injury, xerostomia, and antibiotic use, causing candidiasis, which could potentially progress to deeper tissue invasion and fungemia. *Candida* is isolated in most cases from oral lesions in patients with chemotherapy- or transplant-induced neutropenia and is a considerable contributor to oral and systemic infections in this population [16,17,18].

### 2.4. Oral Biofilms

An oral biofilm is a structured, multispecies microbial community that develops on surfaces within the oral cavity, most notably on teeth and dental restorations. It consists of bacteria, fungi, viruses, and their metabolic products, all encapsulated within a self-produced extracellular matrix composed of polysaccharides, proteins, and nucleic acids [20,21]. Biofilm formation starts with the adhesion of primary colonizers to the salivary pellicle, subsequently followed by co-aggregation of additional microbial species, resulting in a highly ordered and stratified ecosystem [22,23].

Oral biofilms act as reservoirs for these pathogens, serving a protected niche that facilitates persistence, resistance to antimicrobials, and periodical release of organisms into the bloodstream, especially when neutrophil-mediated surveillance is defective. The subgingival biofilm in periodontitis harbors not only classic periodontal pathogens but also medically important organisms such as VGS, *Enterococcus*, *Pseudomonas*, and *Candida*, all of which can contribute to the progression from localized oral infection to bacteremia or sepsis in neutropenic patients [24,25].

## 3. Epidemiology

The literature consistently emphasizes that oral infections are a significant risk in neutropenic patients and can serve as a source for systemic infection, including sepsis, especially when mucosal barrier injury is present. In a cross-sectional study of patients with chemotherapy-induced neutropenia, oral ulceration was noted in 48% of neutropenic patients, with other oral infections (fungal 20%, viral 15%) as well present [26]. Registry data from patients with severe chronic neutropenia illustrate that oral infectious events are common, with 754 oral infectious episodes becoming apparent over 1913 persons [27].

The incidence of oral sepsis (bacteremia or sepsis originating from oral sources) in neutropenic patients is reported to be as high as 67% during the neutropenic phase coming after hematopoietic stem cell transplantation, with viridans group streptococci (VGS) being the most frequently implicated pathogens [10]. The progression typically follows a pathway from gingivitis and ultimately to bacteremia or sepsis, particularly when oral mucosal barriers are compromised by neutropenia and mucositis. VGS, especially *Streptococcus mitis* and *Streptococcus oralis*, are the predominant organisms isolated from blood cultures in neutropenic patients with oral sepsis, and their entry is strongly associated with periodontal inflammation and bleeding on probing [10,12,28]. *Enterococcus* species, *Pseudomonas aeruginosa*, and *Candida albicans* are also detected at high prevalence in the subgingival biofilm of patients with periodontal disease and can contribute to systemic infection in this population [17,18].

Na et al. conducted a multicenter cohort study of neutropenic sepsis, which involved patients with hematologic malignancies. They noticed that neutropenic patients frequently presented with mucosal barrier injury, which included oral mucositis, which served as a portal of entry for pathogens. Clinical characteristics were associated with rapid progression to septic shock (43.6% incidence), higher organ dysfunction scores, and a predominance of Gram-positive bacteremia, often originating from the oral cavity. Risk factors for progression included profound neutropenia, underlying hematologic malignancy, and mucosal barrier impairment. Outcomes were poor, with hospital mortality of 42.2% and early deaths within the first week of sepsis diagnosis, confirming the aggressive course of sepsis in this population [29].

A prospective observational study in allogeneic hematopoietic stem cell transplantation recipients revealed that patients with gingivitis or periodontitis had a pronouncedly higher rate of bacteremia, most of all with oral viridans streptococci and coagulase-negative staphylococci during neutropenia. The study submitted evidence that periodontal infection is a meaningful risk factor for bacteremia in this population, with progression occurring during the neutropenic phase [10].

Another prospective study of 288 episodes of bacteremia in neutropenic cancer patients found a significant increase in Gram-positive bacteremia, especially viridans group streptococci and coagulase-negative staphylococci, with oral mucositis identified as a major source. The study elucidated a definitive association between oral mucosal injury and accelerated progression to bloodstream infection in neutropenic patients [30].

## 4. Common Clinical Manifestations of Oral Infections in Neutropenic Patients

Involve oral ulcerations (usually on non-keratinized mucosa), necrotizing gingivitis and periodontitis (indicated by rapid tissue destruction, pain, and bleeding), periapical abscesses (deep-seated pain, swelling, and occasional fistula development), and mucositis-associated infections (erythema, ulceration, and pseudomembrane development). Fungal superinfections, most frequently due to *Candida* species, present as white plaques, erythematous lesions, or angular cheilitis, while *Aspergillus* can cause necrotic ulcers or palatal/eschar lesions, particularly in severe immunosuppression [26,31] (Table 1).

Clinical signs of infection may be subtle or absent due to impaired neutrophil response, so fever may be the only presenting symptom. Necrotizing periodontal disease can progress rapidly, with minimal local inflammation, and periapical abscesses may lack classic fluctuance or erythema. Oral lesions are frequently colonized or infected with viridans group streptococci, *Enterococcus*, *Pseudomonas*, and *Candida*, which can seed the bloodstream and cause bacteremia or sepsis [10,11].

Sepsis and septic shock from oral sources are well-documented in neutropenic patients, with high morbidity and mortality. Case reports and cohort studies describe rapid progression from dental or periodontal infection to septic shock, multi-organ failure, and death, especially in the context of viridans streptococcal bacteremia. A case report of an 8-year-old girl with leukemia shows she developed acute necrotizing ulcerative gingivitis and subsequent bacteremia due to *Stenotrophomonas maltophilia*, despite appropriate antimicrobial therapy [32]. Another article reports four cases of *Fusobacterium nucleatum* bacteremia in febrile neutropenic patients with hematological malignancy, of which three had oral mucositis or edema of the oral mucosa as the probable entry spot [33]. The rapid debut of bacteremia in these cases highlights the vulnerability of neutropenic patients to systemic infection originating from oral sources, particularly when mucosal integrity is compromised.

## 5. Prevention: Prophylactic Oral Care

### 5.1. Current Guidelines for Dental Clearance Before Chemotherapy/HSCT

Current guidelines recommend that all patients scheduled for chemotherapy or hematopoietic stem cell transplantation (HSCT), especially those at high risk for oral sepsis from pathogens such as viridans group streptococci, *Enterococcus*, *Pseudomonas*, and *Candida*, perform comprehensive dental screening and clearance before therapy initiation. The Multinational Association of Supportive Care in Cancer/International Society of Oral Oncology (MASCC/ISOO) and the Infectious Diseases Society of America both emphasize the need for a careful dental evaluation to identify and manage active oral infections, including necrotizing gingivitis/periodontitis, periapical abscesses, mucositis-associated infections, and fungal superinfections [34,35].

Screening should include a detailed clinical and radiographic dental evaluation to detect acute and chronic oral foci, with prioritization of treatment for active infections and acute pathology. Management should address extraction or classical treatment of teeth with acute infection, drainage of abscesses, debridement of necrotic tissue, and initiation of antifungal therapy for suspected or confirmed fungal superinfections. Non-urgent dental procedures and treatment of chronic, asymptomatic lesions may be postponed until after immune recovery, as supported by recent data [35,36].

**Figure 2:**
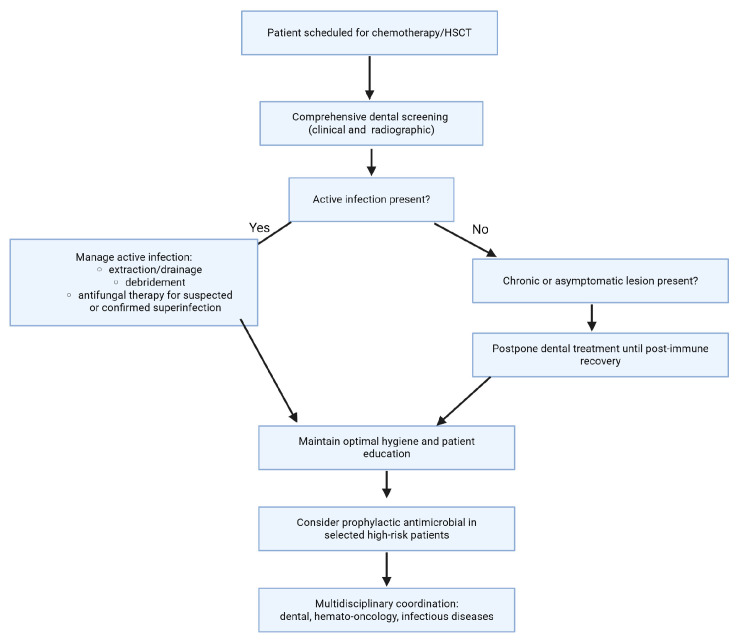
Algorithm for dental risk assessment and management before chemotherapy or HSCT.

Patient education on meticulous oral hygiene and prompt reporting of oral symptoms is vital. Prophylactic antimicrobial regimens targeting high-risk pathogens (e.g., viridans streptococci) may be taken into consideration in select cases, as recommended by the Infectious Diseases Society of America, particularly during profound neutropenia or in the appearance of severe mucositis [4,35]. Close coordination between dental and hemato-oncology teams, while discussing with an infectious disease specialist, represents the key to optimizing timing and minimizing delays in cancer therapy [37]. This strategy lowers the risk of morbidity and mortality from dental sepsis, supports uninterrupted cancer treatment, and improves overall outcomes in this vulnerable population. Figure 2 outlines a structured decision pathway for pre-chemotherapy and pre-HCST, illustrating the steps from initial dental screening to the management of active infections, patient education, and a multidisciplinary approach to reduce sepsis and ensure uninterrupted cancer therapy. (Figure 2).

### 5.2. Timing of Dental Interventions Before Immunosuppression

Current consensus in the medical literature is that dental procedures for the treatment of active oral infections should ideally be completed at least 14 days before initiating immunosuppressive therapy to allow for tissue healing and monitoring for postoperative complications [35,38]. Observational studies and rapid reviews show that dental extractions performed less than 2 weeks before chemotherapy or transplantation increase the risk of complications, including local infections, delayed healing, and, in the case of radiotherapy, osteoradionecrosis [38,39].

For invasive procedures, such as extractions or drainage of periapical abscesses, a 2-week interval is considered minimal to reduce the risk of infectious complications and to ensure the patient’s integration into the oncology protocol without delay. If oncology treatment cannot be postponed, a shorter interval may be acceptable, with careful clinical monitoring, but the risk of complications increases proportionally [40,41]. Interventions on chronic, asymptomatic lesions (e.g., chronic periodontitis without signs of exacerbation) can be postponed until after immune recovery, without significant impact on the risk of severe infections during the period of immunosuppression [36,40]. The pre-therapy dental assessment strategy must be multidisciplinary, necessitating strong collaboration between the oncology and dentistry teams, to optimize the timing of interventions and reduce the risk of oral sepsis [37,41] (Table 2).

### 5.3. Role of Chlorhexidine and Professional Cleaning

Chlorhexidine mouth rinses are extensively used in oral care protocols for patients programmed for chemotherapy or hematopoietic stem cell transplantation (HSCT), but current evidence does not support their efficacy in preventing oral mucositis or reducing the incidence of oral infections in this population. A systematic review found that chlorhexidine did not significantly reduce the incidence or severity of chemotherapy-induced oral mucositis compared to placebo or saline, and it is associated with adverse effects such as tooth staining and taste alteration. As a consequence, routine use of chlorhexidine for mucositis prevention is not recommended [42]. The Multinational Association of Supportive Care in Cancer/International Society of Oral Oncology (MASCC/ISOO) and the European Society for Blood and Marrow Transplantation (EBMT) recommend basic oral care and patient education, but do not improve the position of chlorhexidine as a standard preventive measure [43].

Povidone-iodine rinses have demonstrated some benefit in reducing the severity of oral mucositis in a single randomized trial; however, the evidential foundation is limited and not sufficient for routine recommendation in guidelines [42]. There is no strong certification supporting their use for the prevention of oral infections or complications in the pre-chemotherapy/HSCT setting.

Professional dental cleaning and meticulous dental evaluation are strongly recommended by MASCC/ISOO and the Infectious Diseases Society of America as part of dental clearance sooner than immunosuppressive therapy. These interventions aim to remove active sources of infection, optimize periodontal health, and reduce the risk of oral and systemic complications during neutropenia [34,35,44]. Professional cleaning is a core component of these protocols and is associated with a reduction in the severity and incidence of oral mucositis and infectious complications [45].

### 5.4. Controversies: Prophylactic Antibiotics Before Dental Procedures in Neutropenic Patients

The use of prophylactic antibiotics before dental procedures in neutropenic patients about to undergo immunosuppressive therapy is a controversial subject, with recommendations changing based on patient risk, moment of intervention, and local resistance patterns. The Infectious Diseases Society of America and the American Society of Clinical Oncology recommend that all patients scheduled for chemotherapy or hematopoietic stem cell transplantation (HSCT) undergo comprehensive dental evaluation and management of active oral infections, ideally completed at least 10–14 days before immunosuppression to allow for healing and reduce infection risk [34,35].

Routine use of prophylactic antibiotics, especially for dental procedures in neutropenic patients, is not universally recommended. The Infectious Diseases Society of America and the American Society of Clinical Oncology recommend systemic antibacterial prophylaxis (typically with a fluoroquinolone) only for patients expected to experience profound, prolonged neutropenia (ANC < 500/μL for ≥7 days), and not specifically for dental procedures, but rather for the complete period of neutropenia [4,46] (Table 3). No consensus or high-quality evidence supports additional antibiotic prophylaxis specifically for dental interventions in this setting, and concerns about antimicrobial resistance and adverse effects are significant [47].

For patients with severe neutropenia who demand urgent dental procedures and cannot delay immunosuppressive therapy, some studies consider peri-procedural antibiotics on a case-by-case basis, but this is not a formal guideline recommendation [35]. The choice of agent, if used, should be guided by local resistance patterns and individual risk factors [47,48].

### 5.5. Emerging Strategies

Probiotics have demonstrated efficacy in reducing the incidence and severity of oral mucositis and other oral complications in patients undergoing chemotherapy or hematopoietic stem cell transplantation. Multiple meta-analyses and randomized controlled trials show that probiotic supplementation, particularly with Lactobacillus and multi-strain formulations, lowers in a great measure the risk of oral mucositis (including severe grades) and reduces infection rates and the need for enteral nutrition during cancer therapy. These benefits are associated with anti-inflammatory, immunomodulatory, and microbiome-stabilizing effects, and are supported by recent reviews and systematic reviews [49,50,51]. Probiotic strategies are also associated with a reduction in overall cancer treatment complications, including infections, and are recommended as adjuncts to standard oral care in this context [52,53]. Thus, probiotics have the strongest evidence for reducing oral complications and infection risk in this population, while antimicrobial photodynamic therapy and biomaterial coatings are promising but require further validation before routine clinical adoption.

Antimicrobial photodynamic therapy (aPDT) is an emerging adjunctive modality for the management and prevention of oral infections, in particular in the setting of oral mucositis and biofilm-associated disease. Clinical studies indicate that aPDT, especially when combined with photosensitizers such as curcumin and blue LED, can reduce pathogenic bacterial and fungal loads (for example, *Candida* spp.), decrease the severity of mucositis, and provide analgesic benefits. aPDT has demonstrated potential as an adjunct to routine care, with some studies reporting earlier clinical improvement compared to conventional treatments. But despite that, heterogeneity in protocols and limited high-quality randomized trials preclude routine recommendation, and further research is needed to define optimal parameters [54,55].

Biomaterial coatings, such as chitosan-based formulations, are under investigation for their mucosal protective, anti-inflammatory, and antimicrobial characteristics. Chitosan demonstrates potential as a mucosal barrier and drug delivery system, with proof of reduced mucositis severity and improved healing in preclinical and early clinical studies. These biomaterials may offer supplementary benefit in maintaining oral mucosal integrity during neutropenia, but clinical data are still emerging [56].

## 6. Early Recognition and Rapid Diagnostics

### 6.1. Early Clinical Indicators for Oral Infection During Neutropenia

Initial clinical indicators for oral infection in neutropenic patients encompass subtle or unexplained oral or facial edema, disproportionate discomfort relative to examination results, new or persistent oral ulcers, mucosal erythema, necrosis, or the presence of fever without an identifiable source. In neutropenic patients, classic signs of inflammation may be minimal or absent, so even minor oral findings should prompt concern for infection and rapid diagnostic evaluation. The Infectious Diseases Society of America emphasizes that any skin or mucosal lesion, no matter how small or inoffensive, stands in need of careful evaluation in neutropenic patients, as these may represent early or occult infection [57].

The American Society of Clinical Oncology and the Infectious Diseases Society of America underscore that, in the absence of an alternative explanation, fever in neutropenic patients should be presumed infectious in origin, and an exhaustive history and physical examination should specifically require inspection of the oral cavity for subtle lesions, swelling, or ulceration [1]. Oral infections may present atypically, and the oral cavity, including the periodontium, dentition, and salivary glands, can be an unevaluated source of infection, with symptoms often minimized due to impaired inflammatory response [58].

Early recognition is important, as oral infections can swiftly progress to deep tissue involvement or sepsis in this population. Any of the above findings should prompt microbiologic workup (including blood cultures and, if indicated, lesion swab or biopsy), imaging if deep tissue involvement is suspected, and empiric broad-spectrum antimicrobial therapy [1,57] (Table 4).

### 6.2. Imaging Tools: CBCT and MRI in Immunocompromised Patients

Magnetic resonance imaging (MRI) is the preferred method for early recognition and rapid diagnosis of deep oral and maxillofacial infections in immunocompromised, neutropenic patients, especially when clinical findings are modest. MRI offers superior soft tissue contrast, enabling detection of early cellulitis, abscess formation, and deep fascial space involvement, frequently overlooked on clinical exam or plain radiographs. Cone beam computed tomography (CBCT) is valuable for high-resolution imaging of osseous structures and dental pathology, but is less sensitive than MRI for early soft tissue changes and deep space infections. Conventional CT remains useful for rapid assessment of bony involvement and for surgical planning, but MRI is favored for early, subtle soft tissue infection in neutropenic hosts [59].

### 6.3. Biomarkers

They work together to perform a complementary role in early sepsis detection. C-reactive protein (CRP) is sensitive but nonspecific and may be less reliable in neutropenic patients due to blunted inflammatory responses. Procalcitonin (PCT) demonstrates higher specificity and negative predictive value for bacterial infection and sepsis in neutropenic patients, with area under the curve (AUC) values > 0.75 for sepsis detection in hematologic malignancy. Presepsin is a new biomarker with very high diagnostic accuracy (AUC up to 0.996 in some cohorts), outperforming CRP and PCT in some studies; however, more testing and validation are needed [60,61,62,63].

### 6.4. Rapid Molecular Diagnostics

These methods include polymerase chain reaction (PCR) panels and matrix-assisted laser desorption/ionization time-of-flight mass spectrometry (MALDI-TOF MS). Thus, it enables earlier and broader pathogen identification compared to conventional cultures, which are often negative in neutropenic fever. PCR-based methods can rapidly identify bacterial and fungal DNA from blood or tissue, improving diagnostic yield and enabling targeted therapy, but sensitivity may be lower in neutropenic patients compared to immunocompetent individuals [64,65]. MALDI-TOF MS accelerates identification from positive cultures but is limited by the need for organism growth. Combined molecular and biomarker approaches offer the highest diagnostic accuracy for early infection and sepsis in this population [66].

The American Society of Clinical Oncology and the Infectious Diseases Society of America recommend integrating clinical, imaging, biomarker, and fast molecular diagnostic data for early awareness and management of infection in neutropenic patients [1].

### 6.5. Salivary Diagnostics in Neutropenic Patients

Salivary diagnostics offer a promising, noninvasive approach for early detection of oral infection and sepsis in neutropenic, immunocompromised patients, especially given the challenges of subtle clinical results and the need for rapid assessment. Saliva contains a diverse array of biomarkers, including inflammatory cytokines (e.g., IL-6, IL-8), acute phase proteins (CRP, procalcitonin), and microbial DNA, that reflect both local oral and systemic pathophysiologic changes [67,68,69,70]. Recent studies demonstrate that salivary IL-6 is significantly elevated in patients with sepsis, suggesting utility for early diagnosis in hospitalized populations, including those with neutropenia [71]. Salivary cultures can also correlate with bloodstream infections and antimicrobial resistance patterns, supporting their role in predicting systemic infection in the ICU setting [72]. Advances in point-of-care microfluidic and biosensor technologies have enabled rapid, sensitive detection of salivary biomarkers, facilitating real-time monitoring and early intervention [73,74].

However, clinical implementation is limited by variability in sample collection, processing, and a lack of standardized thresholds for biomarker interpretation [67,70]. While salivary diagnostics are highly attractive for serial monitoring and screening, further validation in neutropenic cohorts is needed to establish diagnostic accuracy and clinical impact.

## 7. Opportunities

Recent advancements offer significant opportunities to move from generalized management toward personalized, precision-based care for oral infections in neutropenic patients. Rapid molecular diagnostics and metagenomic sequencing are revolutionizing the early identification of bacterial and fungal pathogens, facilitating quicker differentiation between colonization and invasive infection, and promoting earlier de-escalation. Colonization screening for resistant organisms is expanding from gastrointestinal regions to oral sites to inform empiric regimen choices. Furthermore, machine-learning risk prediction tools and dynamic antibiograms provide innovative support for real-time, individualized decision making. In dentistry, emerging minimally invasive techniques—such as laser-assisted debridement, photobiomodulation for mucositis, and silver diamine fluoride for caries management—may alleviate infectious burdens while reducing procedural trauma. Together, these advancements underscore a shift towards individualized, risk-adjusted strategies for neutropenic patients.

## 8. Knowledge Gaps

Despite progress, notable knowledge gaps remain. Existing diagnostic criteria for oral infections in neutropenic patients are inconsistent, and the lack of validated severity scales hampers comparative research and standardized clinical decision making. The evidence supporting the timing and safety of dental procedures during cytopenia primarily consists of observational studies, with few prospective investigations assessing optimal thresholds for ANC, platelet counts, or inflammatory markers. There is also a lack of standardized protocols for dental management in patients with congenital or acquired neutropenia, and the efficacy of antibiotic prophylaxis and the use of granulocyte colony-stimulating factor (G-CSF) is not supported by robust clinical trials, and further research is needed [27,58]. Oral symptoms may be underestimated or overlooked, and microbiological documentation of oral infections is often incomplete. Additionally, the interplay among the oral microbiome, mucosal immunity, and pathogen virulence in neutropenia is inadequately understood, especially in relation to biofilm dynamics and fungal-bacterial interactions. The ramifications of antifungal resistance in oral species remain underexplored, with limited data on cross-resistance patterns in patients undergoing extended prophylaxis.

## 9. Conclusions

Oral infections in neutropenic patients present as diverse lesions, with severity linked to the depth and duration of neutropenia. Fungal co-infections, notably *Candida* and *Aspergillus*, significantly cause morbidity. Diagnosis requires a thorough clinical assessment supplemented by microbiological and advanced molecular techniques, including metagenomic methods, for improved pathogen identification. Nevertheless, disparities in diagnostic resources and criteria hinder uniform management practices across institutions.

Evidence indicates the necessity of routine dental assessments before myelosuppressive treatment, with optimal oral disease management concluded 10–14 days before immunosuppression. Clinicians must exercise increased vigilance for atypical oral manifestations during neutropenia, promptly initiate empirical treatment for suspected infections, and adhere to antimicrobial stewardship to mitigate resistance. Effective collaboration among dental, hematology-oncology, and infectious disease professionals is crucial to avert treatment delays and minimize systemic complications such as bacteremia and sepsis. Future investigations should focus on enhancing diagnostic protocols, creating validated risk assessment tools, and elucidating host-pathogen-microbiome dynamics in neutropenia. Further research is essential to understand antifungal resistance trends and to formulate standardized prophylactic and therapeutic guidelines suited to various clinical scenarios.

In the context of advancing supportive care, personalized medicine principles should guide the development of diagnostic algorithms, risk-based dental protocols, and individualized strategies.

## Figures and Tables

**Table 1:** The most common clinical manifestations of oral infections in neutropenic patients, their clinical features, and associated pathogens.

Clinical Manifestation	Typical Features	Microbial Colonization/Infection	Notes in a Neutropenic Setting
Oral ulcerations (non-keratinized mucosa: tongue, buccal mucosa, soft palate)	Painful or asymptomatic ulcers, erythematous halo, impaired healing	Viridans streptococci, HSV reactivation, *Candida*	May be subtle; sometimes fever is the only sign
Necrotizing gingivitis/periodontitis	Rapid tissue destruction, severe pain, spontaneous bleeding, halitosis	*Fusobacterium*, *Prevotella*, spirochetes, *Candida* co-infection	Can progress rapidly with little visible inflammation due to neutropenia
Periapical abscesses	Deep-seated pain, swelling, possible sinus tract or fistula	Viridans streptococci, *Enterococcus*, Gram-negatives	Classic signs (fluctuance, erythema) may be absent
Mucositis-associated infections	Erythema, pseudomembranes, ulceration, secondary bacterial/fungal colonization	Viridans streptococci, Pseudomonas, Candida	A major source of bacteremia during chemotherapy
Fungal infections (*Candida* spp.)	White, removable plaques, erythematous lesions, and angular cheilitis	*Candida albicans*, *C. glabrata*	Common in prolonged neutropenia or after broad-spectrum antibiotics
Fungal infections (*Aspergillus* spp.)	Necrotic ulcers, palatal perforation, black eschar	*Aspergillus fumigatus*, *A. flavus*	Rare but severe; often seen in profound/prolonged neutropenia

**Table 2:** Clinical decision guide that outlines the time dental interventions in patients preparing for immunosuppressive therapy.

Clinical Scenario	Intervention	Timing Before Immunosuppression	Notes
Active oral infection (e.g., periapical abscess, acute periodontitis, cellulitis)	Extraction, drainage, urgent treatment	Ideally ≥14 days before therapy	Shorter interval acceptable if oncology treatment cannot be postponed, but increased risk ↑
Invasive procedures (e.g., extractions, surgery)	Complete before immunosuppression	≥14 days healing	Allows monitoring for complications (infection, delayed healing, osteoradionecrosis in RT)
Chronic asymptomatic lesions (e.g., chronic periodontitis, root remnants without infection)	Deferral	After immune recovery	Low short-term risk; avoid delaying cancer treatment
No infection or significant dental issues	No intervention needed	Proceed with oncology protocol	Needs oral hygiene and preventive care
Oncology protocol cannot be delayed	Perform urgent intervention only	Even <14 days if necessary	Requires multidisciplinary monitoring and prophylactic strategies

**Table 3:** The table summarizing the guidelines shows that the Infectious Diseases Society of America and the American Society of Clinical Oncology recommend against routine prophylactic antibiotics before dental procedures in neutropenic patients about to undergo immunosuppressive therapy.

Guideline Source & Year	Patient Population	Indication for Prophylactic Antibiotics	Recommended Agent(s)	Timing Relative to Dental Procedures	Special Considerations
American Society of Clinical Oncology (ASCO) & Infectious Diseases Society of America (IDSA), 2018	Adults with cancer-related immunosuppression (chemotherapy, HSCT)	Not routinely recommended for dental procedures; recommended only for patients at high risk for profound, prolonged neutropenia (ANC < 500/μL for ≥7 days)	Fluoroquinolone (e.g., levofloxacin) for high-risk patients	Prophylaxis during the entire period of neutropenia, not specifically peri-procedural	Consider local resistance rates, risk of C. difficile, microbiome disruption, and stewardship
Infectious Diseases Society of America (IDSA), 2020 (Pediatrics)	Pediatric cancer and HSCT patients	Not routinely recommended for dental procedures; levofloxacin is preferred for high-risk neutropenia	Levofloxacin (ciprofloxacin alternative)	Restrict to the expected period of severe neutropenia (ANC < 500/μL)	Local resistance epidemiology is critical; adverse effects must be discussed

**Table 4:** Early clinical indicators of infection guide for oral and maxillofacial infections in neutropenic patients, designed to help clinicians recognize early, often subtle warning signs where the usual inflammatory responses may be muted. It pairs each sign with its description, the guideline recommendations (ASCO/IDSA), and the appropriate management and evaluation steps.

Early Clinical Indicators of Infection	Description	Guideline Recommendations (ASCO/IDSA)	Management/Evaluation
Barely visible or unexplained oral/facial swelling	Subtle or disproportionate swelling may lack erythema	Prompt recognition as possible deep infection; diminished signs due to neutropenia	Immediate physical exam, imaging if deep space suspected, rapid empiric antibiotics
Pain out of proportion to exam findings	Severe pain with minimal visible findings	Consider occult infection or necrotizing process	Urgent evaluation, consider imaging, initiate broad-spectrum empiric antibiotics
New or persistent oral ulcers	Ulcers not explained by trauma or mucositis	High suspicion for infectious etiology, especially if persistent or worsening	Swab for culture if feasible, empiric antibiotics, antifungal/antiviral if indicated
Mucosal erythema	Reddened mucosa may be subtle	May be the only sign of infection; do not rely on classic inflammatory signs	Careful inspection, empiric therapy if other signs present
Mucosal necrosis	Areas of tissue breakdown or black eschar	Suggests severe infection, possible invasive fungal or bacterial process	Urgent imaging, surgical consult if necrotizing, immediate broad-spectrum antibiotics
Fever without an identifiable source	Fever in a neutropenic patient, no clear focus	Assume infection until proven otherwise; fever may be the only sign	Rapid assessment, blood cultures, and empiric broad-spectrum antibiotics within 1 h of presentation
Afebrile or hypothermic with concerning oral findings	Infection may present without fever in profound neutropenia	Do not exclude infection based on the absence of fever	Treat as an infectious emergency if early clinical signs of infection, empiric therapy

## Data Availability

No new data were created or analyzed in this study.

## Abbreviations

The following abbreviations are used in this manuscript:

ANC	Absolute Neutrophil Count
HSCT	Hematopoietic Stem Cell Transplantation
CAR	Chimeric Antigen Receptor
VGS	Viridans Group Streptococci
HSV	Herpes Simplex Virus
PISA	Periodontal Inflamed Surface Area
CRP	C-Reactive Protein
PCT	Procalcitonin
AUC	Area Under the Curve
PCR	Polymerase Chain Reaction
MALDI-TOF MS	Matrix-Assisted Laser Desorption/Ionization Time-of-Flight Mass Spectrometry
IL-6	Interleukin-6
IL-8	Interleukin-8
CBCT	Cone Beam Computed Tomography
MRI	Magnetic Resonance Imaging
CT	Computed Tomography
RT	Radiotherapy
MASCC/ISOO	Multinational Association of Supportive Care in Cancer/International Society of Oral Oncology
IDSA	Infectious Diseases Society of America
ASCO	American Society of Clinical Oncology
EBMT	European Society for Blood and Marrow Transplantation
aPDT	Antimicrobial Photodynamic Therapy
LED	Light-Emitting Diode
ICU	Intensive Care Unit
AMS	Antimicrobial Stewardship
ESBL	Extended-Spectrum Beta-Lactamase
MDR	Multidrug-Resistant
DNA	Deoxyribonucleic Acid
AI	Artificial Intelligence
